# Cell Lines for the Development of African Swine Fever Virus Vaccine Candidates: An Update

**DOI:** 10.3390/vaccines10050707

**Published:** 2022-04-29

**Authors:** Dionigia Meloni, Giulia Franzoni, Annalisa Oggiano

**Affiliations:** Department of Animal Health, Istituto Zooprofilattico Sperimentale della Sardegna, 07100 Sassari, Italy; dionigia.meloni@izs-sardegna.it (D.M.); annalisa.oggiano@izs-sardegna.it (A.O.)

**Keywords:** ASFV, cell line, adaptation

## Abstract

African swine fever virus (ASFV) is the etiological agent of a highly lethal disease in both domestic and wild pigs. The virus has rapidly spread worldwide and has no available licensed vaccine. An obstacle to the construction of a safe and efficient vaccine is the lack of a suitable cell line for ASFV isolation and propagation. Macrophages are the main targets for ASFV, and they have been widely used to study virus–host interactions; nevertheless, obtaining these cells is time-consuming and expensive, and they are not ethically suitable for the production of large-scale vaccines. To overcome these issues, different virulent field isolates have been adapted on monkey or human continuous cells lines; however, several culture passages often lead to significant genetic modifications and the loss of immunogenicity of the adapted strain. Thus, several groups have attempted to establish a porcine cell line able to sustain ASFV growth. Preliminary data suggested that some porcine continuous cell lines might be an alternative to primary macrophages for ASFV research and for large-scale vaccine production, although further studies are still needed. In this review, we summarize the research to investigate the most suitable cell line for ASFV isolation and propagation.

## 1. Introduction

African swine fever virus (ASFV) is the causative agent of African swine fever (ASF), a highly hemorrhagic contagious disease of swine that requires notification to the World Organization for Animal Health (OIE) [1]. This virus was discovered in Kenya a century ago [2] and is now—in the second decade of the 21st century—the cause of a pandemic in pigs (*Sus scrofa domestica*), wild boars (*Sus scrofa*), and other free-ranging suids [3]. The etiological agent is the ASF virus (ASFV), a large double-stranded DNA virus belonging to the family *Asfaviridae*.

Twenty-four distinct ASFV genotypes have been identified based on phylogenetic analysis of a fragment of the variable region of the B646L gene, encoding for the major protein p72 [4,5]. All genotypes are endemic in Africa, and only two (I and II) have spread around the world [6,7,8]. It is considered a major threat to the worldwide swine industry, as it is characterized by a fast transcontinental spread, and it is currently present in Africa, Europe, Asia, and Oceania and was most recently introduced in the Americas [1,8]. 

Strict biosecurity protocols and classic sanitary methods both at the farm and at national levels have been adopted to fight the spread of this virus; however, these strategies are remain insufficient, and in some countries they are difficult or impossible to follow owing to the lack of available resources [9,10,11,12]. As there are no licensed vaccines or treatments available, the most important control strategies for ASFV are early virus detection, followed by laboratory diagnosis [13], and the subsequent culling of ASFV-infected animals from the herds to prevent the spread of the virus [14]. 

However, the biological properties of ASFV indicate that this pandemic will continue to develop, and the development of an effective and safe vaccine would be the best solution to contain epidemics and reduce the significant economic losses related to ASF outbreaks [3]. In the last 40 years, various vaccination strategies have been attempted in search of an optimal vaccine; however, the complexity of the virion composition, its life cycle, and its ability to evade the host’s immune system have created many difficulties [3,15,16,17]. Furthermore, another important obstacle that has limited the construction of a safe and efficient vaccine is the lack of a suitable continuous cell line for ASFV isolation and propagation [3,18]. 

Although monocytes and macrophages are the main targets of ASFV infection [19], the virus can also replicate in established cell lines with less efficiency [18,20]. Macrophages have been widely used to study virus–host interactions, and we and others have observed that these cells respond differently to infection by virulent and attenuated ASFV in vitro [21,22]. Although the use of these primary cell lines offers some advantages, the generation of primary macrophages is time-demanding and expensive, and contamination may occur, leading to cell wastage [4]. 

In addition, the use of primary cells from animals for large-scale vaccine production is not practical, and it is also challenging from an ethical point of view. To overcome these issues, numerous efforts have been made to create an established immortalized cell line of macrophages to improve investigations of ASFV biological properties (i.e., the replication cycle, host immune modulation, and pathogenesis) to design specific diagnostic systems, antiviral compounds, and efficient vaccine candidates [23].

We need to discover the mechanisms beyond ASFV replication in different cell lines in order to develop cell lines more suitable for ASFV field isolate replication. Identification of the ASFV proteins that induce protective immune responses is the major challenge to developing an efficient ASF vaccine on a large scale [3,15,17].

Within this review, we elucidate the current characteristics, advantages, and disadvantages of different cell lines used for the isolation, propagation, titration, and genetic manipulation of several ASFV strains. We also hope to outline areas for future studies on a continuous cell line, which are needed to better understand the biological properties of the virus and to sustain large-scale production of a vaccine against ASFV.

## 2. Porcine Macrophages: The Main Target of African Swine Fever Virus

Since the first ASFV studies, it has been well-known that the virus infects and replicates in diverse monocyte–macrophage populations of several organs of pigs (spleen, lymph node, lung, and kidney) [19,24,25]; however, infection in lymphocytes has not been observed [26]. This virus can also replicate in other cell types, such as endothelial cells, hepatocytes, renal tubular epithelial cells, and neutrophils [24,27,28], but monocytes–macrophages are the main target of ASFV infection in both domestic and wild swine [29]. For many years, these cells have been used to isolate ASFV from field outbreaks [30]. Monocytes–macrophages can be isolated from blood and specific organs/tissues, and in past decades, several modifications have been adopted to increase the yield, specificity, and purity of macrophages; nowadays, standardized protocols are available [30].

### 2.1. African Swine Fever Virus Entry and Receptor

The ASFV infectious cycle starts with the viral entry into the host cell. The virus can invade the host cells by clathrin-mediated dynamin-dependent endocytosis or other mechanisms, such as phagocytosis and macropinocytosis [31]. A crucial role in virus entry, in either macrophages and VERO cells, is played by cholesterol. Researchers observed that inhibitors of this molecule reduced ASFV internalization into its host cells, and they discovered that ASFV’s ability to enter into macrophages was remarkably dependent on both cholesterol and the lipid raft organization [32].

After internalization, the virus particles are trafficked from the membrane to the center of the cell through the endocytic pathway, and ASFV gradually reaches mature endosomal compartments where it removes the outer shell and inner envelope [31]. The viral genome is then released into the cytoplasm where viral replication takes place, although a nuclear step also occurs [33].

To date, the cell membrane receptor(s) and attachment factor(s) involved in viral entry remain unidentified [31]. In the past, researchers speculated that permissiveness to ASFV infection was correlated to expression of the scavenger receptor CD163, which is restricted to cells of monocyte/macrophage lineage and is a hallmark of macrophage maturation [34]. Researchers observed that the susceptibility of host cells to ASFV was correlated to maturity of porcine monocyte–macrophages, which correlates with an upregulation of CD163 expression [35]. In addition, they showed that monoclonal antibodies against CD163 reduced the efficiency of ASFV to infect monocyte-derived macrophages (moMФ) [35].

Nevertheless, contrasting results have been reported regarding the role of CD163 in ASFV infection. In a recent study, the gene coding for CD163 was deleted from the pig genome using the CRISPR/Cas9 system. Pigs lacking CD163 showed no differences in the course of infection or survival compared to wild-type CD163-expressing pigs [36]. This result indicated that CD163 alone is not sufficient for virus infection, suggesting that other proteins, receptors, or entry mechanisms are also involved in this process [18,31]. To date, scant data are available on the factors influencing ASFV ability to enter and replicate into its target cells. 

We and others previously observed that both type I and type II interferons (IFNs) inhibited ASFV ability to infect macrophages [21,22], whereas other cyotokines, such as TNF-α, promoted this virus ability to replicate into myeloid cells [37]. More recently, it was observed that an inhibitor of the nuclear factor-κβ (NF-κβ) inhibited ASFV replication into porcine alveolar macrophages, suggesting that the NF-κβ pathway is crucial for the ASFV infectious cycle [38]. Future studies in this research are need to better understand the complex interaction of ASFV with its target cells.

### 2.2. Porcine Monocyte and Derived Macrophages

As previously reviewed, the susceptibility of myeloid cells to ASFV seems to be linked to maturity, and in vitro differentiation of porcine blood monocytes into macrophages has been linked to increased susceptibility to ASFV infection [35,39,40,41]. Blood-circulating monocytes can be differentiated into monocyte-derived macrophages (moMФs) by culture in media supplemented with either autologous plasma, M-CSF, or GM-CSF [41,42]. We observed that differentiating conditions (different concentrations of autologous plasma or human M-CSF) did not influence the moMФ susceptibility to virulent 22653/14 ASFV [41].

Although the use of human M-CSF to generate moMФs likely provides a better reproducibility between experiments, ASFV diagnosis is usually performed on moMФs generated using autologous plasma. The presence of infectious ASFV in tissue samples can indeed be identified using the Malmquist test, where tissue homogenates are added to fresh, two-day-old monocyte/macrophage monolayers [43]. The presence of infectious ASFV is generally confirmed by hemadsorption, although the presence of non-hemadsorbing isolates (such as NH/P68 or OUTR88/3) needs to be investigated through immunofluorescence [44].

MoMФs are widely used to study ASFV isolated from field outbreaks and to study virus–host interaction [30]; however, it is difficult to knock out or overexpress the host molecules in primary cells to study the ASFV–host interactions [45]. As stated above, working with these primary cells involves drawbacks, such as ethical constraints (animal welfare considerations) and high time consumption; thus, they are not likely to be used for the large-scale production of ASFV vaccines [18].

### 2.3. Bone-Marrow-Derived Macrophages

It has been reported that porcine bone marrow cells could support ASFV replication [43], although lower levels of infection were observed in the fresh lineage compared to mature alveolar macrophages [46]. The protocol to obtain bone-marrow-derived macrophages was recently described in detail by Lithgow et al. (2014). In brief, cells were first rinsed from femur bones, diluted in PBS, layered over 1077 Histopaque (Sigma, USA), and centrifuged to obtain buffy coat cells. 

After several washings, these cells were resuspended in Earle’s saline plus 10% porcine sera (PS) supplemented with penicillin and streptomycin, and seeded in plastic flasks or plates. Non-adherent cells were removed after 2 h, while the others were cultured for a further 6–7 days [46]. Then, bone-marrow-derived macrophages were infected with ASFV: these cells were susceptible to either virulent or attenuated ASFV strains Benin 97/1, Attenuated Uganda, and Virulent Uganda [46].

Chen and colleagues, in a recent study, showed that the use of primary porcine bone marrow cells supported the growth of HLJ/18-7GD, an ASFV candidate vaccine with seven genes deleted. The attenuated ASFV strain maintained its immunogenicity after being serially passaged on these cells for six passages, suggesting that the use of porcine bone marrow cells was feasible and cost-effective for the large-scale production of HLJ/18-7GD [47].

### 2.4. Porcine Alveolar Macrophages

Porcine alveolar macrophage (PAMs) can be obtained from swine pulmonary lavage using a technique originally adapted to pigs from rabbits [48]. Thus far, these cells have been widely used in ASFV studies. These monolayer cultures, even after thawing, can be directly used for virus titration or for biochemical and immunological studies, giving the same results as those obtained with moMФs [30]. In addition, it was observed that PAMs were more susceptible to ASFV infection in comparison to two other more immature myeloid cells: bone marrow cells and blood monocytes [35].

The advantage of working with PAMs is the greater reproducibility of results compared with using a conventional primary culture because multiple experiments can be performed using the same cell stock [30]. Despite the many advantages of PAMs, working with these primary cells includes some disadvantages, such as high time consumption, expensive cell extraction, and animal welfare considerations [18]. In addition, other issues are the high variation hindering consistent macrophage phenotypes among different pigs and the low reproducibility from day to day [49,50].

### 2.5. Porcine Renal-Derived Macrophages

Macrophages can be efficiently isolated from primary porcine kidney cell cultures [51], and Oh and colleagues recently characterized these cells as renal-derived macrophages presenting the molecular and morphological characteristics of monocyte/macrophage lineage, with high continuity and reproducibility. In detail, immunocytochemistry analyses revealed that these cells expressed macrophage markers CD163, CD172a, and Iba1 [50], indicating that they were a mature macrophage population, and most importantly, that they were susceptible to infection with both wild-type (MW039157) and cell-adapted (MW287337) ASFV genotype II. 

The phenotype of these cells remained consistent for at least two months in different batches, and overall data suggested that these primary renal-derived macrophages would be a suitable alternative to PAM or moMФ [50]. In addition, researchers observed that both strains were efficiently replicated in these cells (using an MOI of 0.3). These cells were also more permissive to the wild-type virus than the continuous cell line MA-104 (see Section 4.3), suggesting that they can be used for ASFV isolation, propagation, and production [50]. Although more studies are needed to evaluate whether the genome and immunogenicity of the virus isolates change after several passages in renal-derived macrophages, these cells might be an alternative cell line for the large-scale production of modified live ASF vaccine [50].

## 3. Other Porcine Primary Cell Lines

### 3.1. Dendritic Cells

Dendritic cells (DCs) are a heterogeneous population that plays a central role in immune responses against pathogens [52]. Despite their important role in innate immunity against foreign invaders, there remains a paucity of studies investigating the interaction of the virus with DCs from blood or from different tissue sites, as we recently reviewed [53].

ASFV can infect skin-derived DCs [54] and DCs in several tissues (the lungs and liver and, at low frequencies, the spleen and lymph nodes) [55]; however, there is a paucity of studies investigating the interaction of the virus with DCs from blood or from different tissue sites [54].

Owing to the low frequency of these cells in blood and tissues, myeloid DCs can be generated in vitro by culturing monocytes in a medium supplemented with recombinant GM-CSF and IL-4 (monocyte-derived dendritic cells, and moDCs) [56]. We and others previously observed that either virulent, low virulence, or avirulent strains of ASFV can infect moDCs [21,57].

Nevertheless, similar to macrophages, obtaining DCs is laborious and expensive, and it is not feasible to use these cells for large-scale vaccine production. Thus, it is likely that in future these cells will be generated only to better understand ASFV–host interaction.

### 3.2. Endothelial Cells

Although macrophages represent the main target of ASFV, the virus can also replicate in endothelial cells, which appear to play a key role in the hemorrhagic pathogenesis [58]. Vallée et al. (2001) performed an in vitro study of ASFV interaction with primary cultures of porcine aortic endothelial cells (PAECs) and bush pig endothelial cells (BPECs), aiming at understanding the role of these cells in the hemorrhagic pathogenesis of ASF disease [59]. 

They obtained PAECs from the aorta and minor vessels of mini pigs, and these cells expressed the angiotensin-converting enzyme (ACE), which is a specific marker for the endothelium [59]. In that study, researchers observed that a highly virulent ASFV strain (Malawi Lil 20/1) productively infected and replicated in these cultures. In addition, the replication kinetics of the virus (Malawi Lil 20/1, dose 10^4^ HAD50/mL) in PAECs, BPECs, and PAMs were similar [59]. 

The authors observed that the virus downregulated MHC class I expression induced by IFN-α, suggesting that it developed a strategy to elude immune detection, to avoid recognition. These results were mainly focused on analysis of the central role of PAECs and BPECs in the hemorrhagic pathogenesis of ASF disease [59], but these cells did not have practical advantages when compared to other porcine primary cell cultures, unless they could be established as immortalized cell lines [60]. Furthermore, primary porcine endothelial cells do not have a long life span in culture, and after four to five passages, they tend to differentiate, reaching senescence [60].

## 4. Monkey-Derived Continuous Cell Lines

As stated above, obtaining primary macrophages is time-demanding and expensive, and contamination may occur, leading to cell wastage [4]. In addition, the use of primary cells from animals for large-scale vaccine production is not practical, and it is ethically challenging. These problems have been relatively overcome by the adaptation of different virulent field isolates to grow on monkey kidney cells, such as Vero, MS, and COS, which are used for most biochemical studies, or other continuous cell lines. Adaptation through several culture passages often leads to generation of ASFV strains capable to replicate well in the corresponding continuous cell lines.

However, these strains usually exhibit decreased virulence in swine, significant genomic alterations, and reduced immunogenicity [15,61,62,63,64]. Phenotypic changes and genome mutations due to adaptation are not well understood; thus, elucidating the mechanisms of cell culture-adapted ASFV replication in cell lines is required to develop cell lines that are more suitable for ASFV field strain replication. The features, advantages, and disadvantages of monkey-derived continuous cell lines used for ASFV in vitro studies are summarized in Table 1.

### 4.1. Vero Cells

Vero cells are an established cell line derived from the African green monkey kidney, frequently used as a model for in vitro ASFV infection [17]. In 1971, Hess reported the adpatation of two field isolates (an ASF virus isolated in Italy and the Tengani isolate from Malawi) to grow on this cell line [65], and in 1976, Enjuanes and colleagues successfully adapted another ASFV isolate to Vero cells (BA71V) [66]. The adaptation of some ASFV isolates to these established cells allowed the use of plaque formation assays to determine viral titers [65,66], and Vero cells have also been used to aid basic virology research in biochemical and structural studies of ASFV [20].

**Figure 1:**
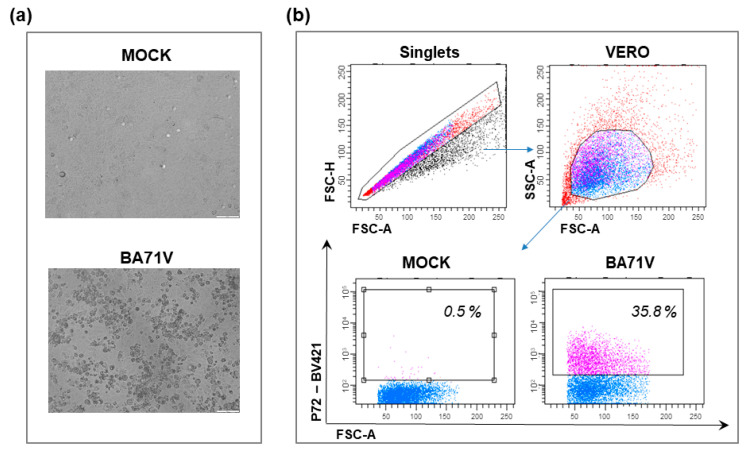
BA71V infection of VERO. VERO cells were infected with the avirulent BA71V ASFV strain using an MOI of 2, alongside mock-infected controls. At 48 h pi, the cytopathic effect was observed with microscopy (**a**) and percentages of ASFV late protein p72^+^ cells were assessed using flow cytometry (**b**). In panel a, representative images of mock-infected and BA71V-infected VERO cells are presented; scale bar 100 μM. In panel b, the gating strategy and representative dot plots are displayed to demonstrate the p72 labeling of VERO cells infected with BA71V ASFV.

The avirulent BA71V, derived by adaptation of the virulent BA71 to replicate in Vero cells [66,67], has been widely used to study the functions of several ASFV genes, the proteomic analysis of the virus, viral transcription, replication, caspase activity, induction of apoptosis, DNA repair, and morphogenesis [68,69,70,71]. It was also used to better understand the ASFV entry into the cell [72,73,74]. Representative images of Vero cells infected with the avirulent BA71V are displayed in Figure 1.

BA71V and Vero cells were also used to screen in vitro antiviral drugs against ASFV, such as several rigid amphipathic fusion inhibitors [75], plant-derived compounds, such as genistein or genkwanin [76,77], and some antibiotics, such as fluoroquinolones [78].

Nevertheless, while ASFV adapted to Vero cells replicated high titers in these cells, they progressively lost their ability to replicate in primary swine macrophages in vitro [20,61,66]. Researchers observed that this progressive adaptation of virulent ASFV isolates to Vero cells was accompanied by either reduced virulence or immunogenicity [61,79], and by significant genomic alterations, such as the appearance of point mutations and deletion, mainly within the left variable ends of the ASFV genome [61,67,80]. 

BA71V was indeed completely avirulent in swine and did not confer protection against challenge with parental BA71 [81]. Another Vero-adapted strain is ASFV-G/V, originated in the adaptation of the ASFV field isolate from the Republic of Georgia (ASFV-G) in Vero cells (110 passages). ASFV-G/V lost multiple multigene family (MGF) genes during adaptation and was fully attenuated in pigs; it also lacked the capacity to protect against challenge with virulent parental ASFV-G [61].

Vero cells are unlikely to be used for the large-scale production of ASFV vaccines because of the progressive genetic mutations of the passaged strain, which results in loss of immunogenicity and the ability to protect against wild-type challenge [61]. Further studies should be performed to elucidate the mechanism of cell-culture-adapted ASFV replication in cell lines, the associated phenotypic changes, and genome mutations.

### 4.2. COS Cells

COS cells, more precisely, COS-1 and COS-7, are other established cell lines derived from the African green monkey kidney that showed the ability to sustain in vitro replication of ASFV and have been used for inexpensive, easy, and reproducible methods to detect and quantify ASFV [20,82]. After evidence of their susceptibility to many different ASFV strains, they have been routinely used for the detection and amplification of many ASFV samples [20,82,83], for the construction of deleted ASFV mutants [84,85,86], for the production of a large amount of virus [20], and for the growth and titration of natural and laboratory-engineered ASFV strains [20,82].

**Figure 2:**
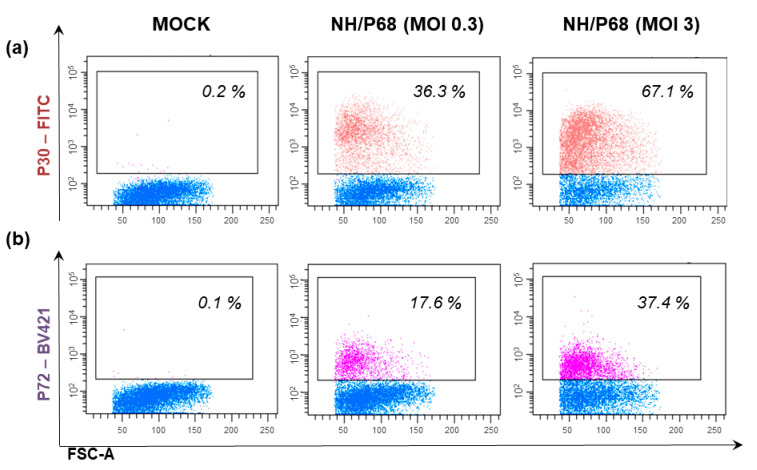
NH/P68 infection of COS-1. COS-1 cells were infected with the low virulence NH/P68 ASFV isolate using an MOI of 0.1 or 1, alongside mock-infected controls. At 24 h pi, the percentages of ASFV early protein p30 (**a**) or late protein p72 (**b**) cells were assessed by flow cytometry. Representative dot plots are displayed to demonstrate the p30 and p72 labeling of COS-1 infected with different MOI of NH/P68 ASFV.

COS-1 cells showed permissiveness to infection with several ASFV isolates, ranging from the virulent España70 (E70), Malawi 82, Uganda virulent strain, Lisbon57, Lisbon60, Mozam 68, and CC83, to the moderately virulent Brazil 81, to the attenuated BA71V, NH/P68, and ΔEP153R [82]. Representative images of COS-1 cells infected with attenuated NH/P68 are presented in Figure 2. 

Moreover, the production of the infective progeny virus was investigated by titration on this cell monolayer, demonstrating that the plaques developed in COS-1 were similar to those observed in Vero cells [6]; thus, these cells became the best choice for ASFV plaque titration [82] and the most appropriate cell system in chromogenic plaque-purification technologies [30]. A plaque assay of ASFV in COS cells was used for the selection and quantification of the infective virus, and purification of virus recombinants was engineered with selectable marker genes [82].

The susceptibility of this line was also compared to that of other cell lines, such as swine macrophages, IPAM, and WSL: cells were infected with 10 different ASFV strains of different origins, and degrees of virulence were screened using a multiplicity of infection (MOI) of 3. All the isolates induced a productive infection in both COS-1 and WSL cells; however, the latter did not develop a clear cytopathic effect; thus, they could not be used for titration of all the tested isolates. COS-1 was able to sustain the growth of BA71V and ΔEP153R isolates, which did not induce a productive infection in swine macrophages (the production levels were almost 10^3^ pfu/mL) [30].

COS-1 was used to investigate the entry mechanism of ASFV into cells, demonstrating that the virus enters via both constitutive macropinocytosis and clathrin-mediated endocytosis [87], and to investigate cellular structures during the infection and intracellular virus trafficking [88].

As stated above, these cells were used for the construction and production of deleted ASFV mutants, including live attenuated vaccines. The attenuated BA71ΔCD2 strain, a genetically modified virus derived from BA71 lacking the CD2V gene, showed remarkable stability when passaged in COS-1 cells: after 20 passages in COS-1 cells, no significant changes were observed in its genome [81].

In addition, it was observed that its parental strain (the virulent BA71 virus) was passaged in COS-1 without loss of stability or integrity in its genome [81], whereas its passage in other continuous cell lines (Vero cells or MS cells) resulted in a large deletion or mutation in its genome [61,63,67]. In addition, BA71 grown in COS-1 maintained its virulence and immunogenicity when pigs were inoculated [81].

COS-7 cells (an SV40T-antigen-transformed epithelial green monkey cell line) were more recently used to generate ASFV live attenuated vaccines (LAVs) [89]. Four live attenuated vaccines based on the low-virulence NH/68 were engineered using COS-7. The authors observed that passage in COS-7 cells provoked changes in the recombinant viruses that were relevant to protection. 

The authors hypothesized that debris from COS-7 might have acted as an immunomodulator; thus, recombinant viruses were purified by Percoll to eliminate the COS-7 cell debris: purified deletion mutants had higher protectivity compared to the unpurified candidate LAV, indicating that the cells and the purification steps played a role in the immunogenicity of the recombinant strain [89]. Overall, COS cells can be efficiently infected by several ASFV isolates and are able to sustain their growth, making them a suitable candidate cell line for the development of a LAV [15]. However, more studies are needed to better understand the behavior of ASFV strains generated from COS cell lines before using them in large-scale vaccine production [15].

### 4.3. MS Cells

MS is a stable cell line derived from the African green monkey kidney, to which some ASFV isolates were adapted to grow and replicate [63]. MS cells did not produce the plaques after ASFV infections [62] that were observed in Vero or COS cells, making this cell line less suitable to titrate ASFV isolates.

Passage of ASFV isolates in this cell line led to large deletions or mutation in the virus’ genome. For example, the virulent strain ASFV E70 was adapted to grow on MS, and viral DNA subpopulations were produced during E70 passage in this cell line [62]. In detail, viral DNA subpopulations were detected after 44 passages in MS but not during the first 14 passages or in the unadapted parental ASFV E70 strain [62]. Different viral variants were isolated, and their genomes were characterized. After 44 passages (E70 MS44), the virus presented deletions that were compared with the wild-type parental strain (E70) [63]. 

The E70 MS44 genome lost almost 17 kbp compared to E70 (the genome was reduced from 173 to 156 kb), mainly because of two deletions at the left and right terminal fragments (a 15.2 kbp deletion located near the left terminus and a 2.4 kbp deletion located near the right terminal fragment), plus two additions of 300 bp in the terminal fragments and a 100 bp addition in the internal Sma I-H1 fragment [63]. In the same study, researchers observed that the central region of the ASFV genome was highly conserved [63]. 

In 1992, Alcaraz and colleagues analyzed the changes in the protein pattern induced by the E70 virus after its adaptation and propagation in MS cells, after 14, 44, and 81 passages. They observed that the selected viral clones at passages 44 and 81 produced a specific protein p54 with a higher molecular weight compared to the wild-type p54 [90]. Moreover, it was observed that while the viral titers increased during its adaptation in MS cells, at the same time the adapted strains, from passage 44 to 81, significantly lost the ability to grow on pig macrophages, demonstrating that many passages in MS cells are needed for ASFV to lose the ability to infect macrophages in vitro [90].

### 4.4. CV1 Cells

The CV1 cell line is a continuous cell line derived from the African green monkey kidney and used for many biochemical, biological, and structural studies of ASFV [20,30] and for titration (performed by plaque assay) [20].

Several ASFV isolates were passaged in this cell line. Ruiz and colleagues adapted España75 (E75), a Spanish ASFV isolated in 1975, in CV1 cells. Interesting, after only four consecutive passages in this cell line, the passaged strain (E75CV1) presented a decreased virulence in vivo compared to the wild type and, most interestingly, protected pigs against the homologous E75 virus challenge [91]. These studies were later extended by Lacasta and collaborators, who observed that all tested pigs survived injection of 10^4^ HAU of E75CV1 (IM route), with only limited viremia and slightly transient fever, whereas the same dose and route of the parental E75 resulted in severe ASF clinical signs starting at 3–5 days pi. 

In addition, researchers observed that the attenuated strain conferred 100% protection against challenge with homologous parental E75: all animal survived infection and no ASF clinical signs, viremia, or nasal viral excretion was detected in any of the injected pigs [92]. Nevertheless, immunization with E75CV1 resulted in a lack of protection against the heterologous BA71 lethal challenge. Further studies are needed to better understand the mechanisms involved in protection against homologous and heterologous lethal challenges of this attenuated strain [92].

The CV1 cell line was also tested by a Russian group: the ASFV strain Stavropol 01/80 (Stavropol) was passaged in this cell line. No cytopathic effects were observed during 20 passages in CV1, although virus presence in these cells was detected by immunofluorescence. When pigs were inoculated with the cultural virus of CV1 passage 20, it was observed that the virus lost pathogenicity; nevertheless, it was unable to confer protection against challenge with a virulent parental strain [93].

### 4.5. Marc-145

Marc-145 is a cell line derived from the African monkey kidney epithelial cell MA-104, and it is permissive to the monocytropic porcine reproductive and respiratory syndrome virus (PRRSV) [94]. It was recently tested for its permissivity to ASFV. Do and Nguyen, in their preliminary study, declared that Marc-145 could be considered a potential cell line for ASFV replication, yet only three passages were monitored [95]. 

On the other hand, Woźniakowski and colleagues tested the ability of Marc-145 to support the growth of the ASFV Pol18/28298/Out111: the strain was passaged 10 times, and its failure to grow on this cell line was revealed by both real-time PCR and an immunoperoxidase test (IPT) [96]. Another study later confirmed the inability of Marc-145 to maintain ASFV replication; an ASFV-HLJ/18 strain was passaged in these cells, and no ASFV genome copy numbers were detected at passage 5 [45].

### 4.6. MA-104 Cells

MA-104 is a commercial cell line that originated from kidney epithelial cells derived from the African green monkey *Cercopithecus aethiops* [97]. Its susceptibility to ASFV and its ability to support viral growth was recently tested by several groups [50,97,98].

In 2020, Ray et al. reported that MA-104 was a suitable substrate for ASFV isolation. Researchers observed that this cell line could detect ASFV (belonging to diverse genotypes) with a TCID_50_ sensitivity comparable to that of primary swine macrophages. In addition, hemadsorption was visible after adding suspension of pig erythrocytes: at 24 h pi with ASFV-G or BA71 these cells presented rosettes similar to those detected on macrophages [97]. This cell line could also detect non-hemoadsorbing strains: at 24 h pi, immunofluorescence was performed (using a monoclonal antibody against early ASFV protein p30) and a clear positive staining was observed [97].

Some preliminary reports from Kwon and colleagues showed that MA-104 was susceptible to infection with genotype II ASFV. In addition, MA-104 cells were used to evaluate the progressive adaptation of ASFV; these cells were able to support the growth of genotype II ASFV isolates and after serial passages (P0, P1, P5, P10, P15) no mutations were detected at the sequences encoding the p72, p54, and p30 proteins [98]. Researchers speculated that MA-104 might be a strong candidate cell line for the development of an ASFV commercial vaccine [98].

Recently, Oh et al. (2021) compared MA-104 to the renal-derived swine macrophages; they observed that MA-104 is less able to isolate the field virus, although both wild-type (MW039157) and cell-adapted (MW287337) ASFV genotype II strains grew efficiently in this continuous cell line [50]. This cell line was also efficiently used to study the interaction network of ASFV with host proteins [99] and to determine the mechanisms used by ASFV to evade the host innate immune responses [100].

Overall, MA-104 discovery could be an opportunity to improve diagnostic capacity and avoid the requirement of donor swine, the latter being time-consuming and unavailable in some laboratories [101]. Nevertheless, more studies are still required to verify whether MA-104 can be considered the best substrate to grow ASFV strains for large-scale vaccine production.

## 5. Human Continuous Cell Lines

### HEK293T Cells

HEK293T is a human embryonic kidney cell line whose susceptibility to ASFV was recently tested [45,102]. HEK293 cells were susceptible to infection with the attenuated OUTR88/3, although their ability to sustain the growth of this isolate was 16-fold lower compared to WSL cells (see Section 6.2) [102]. More recently, these cells were infected by ASFV-HLJ/18, and their ability to support viral growth was evaluated by real-time PCR (to estimate ASFV genome copy numbers), immunofluorescence staining (to monitor ASFV protein expression), and cytopathic effect (CPE) [45]. ASFV-HLJ/18 infected HEK293T cells and replicated at a low level; however, after continuous passaging the strains adapted to these cells and efficiently replicated. 

The ASFV adapted strain was also able to replicate in Vero cells but progressively lost its ability to replicate in PAMs compared to the wild-type strain [45]. The genetic variations acquired by adaptation of ASFV in HEK293T were investigated, and NGS showed that after 121 passages the adapted strains presented several deletions in the last variable region: the total deleted genome corresponded to 25 kb in length in the left end, including 22 MGF genes. Most of the lost MGF genes were associated with virulence pathogenesis, suggesting that ASFV-P121 may be attenuated in pigs, although its pathogenicity and immunogenicity still need to be evaluated in vivo [45].

Features, advantages and disadvantages of this human-derived continuous cell lines used for ASFV in vitro studies are summarized at the end of Table 1.

**Table 1:** Origin and characteristics of monkey-derived and human-derived continuous cell lines used for ASFV in vitro studies.

Cell Line	Species of Cell Origin	Tissue of Cell Origin	Mechanism of Immortalization	Susceptibility to Field Isolates	Susceptibility to Adapted Isolates	Advantages	Disadvantages
**VERO**	African green monkey: *Chlorocebus sabaeus* [103]	Kidney epithelial cells [103]	Spontaneous, unknown process [104]	Low susceptibility to virulent isolates (BA71, Tangani, Hinde, Huganda, Lisbon60) [61,65,66,80]	- Adapted strain from Tengani [65] - BA71V [66,67] - ASFV-G/V [61] - Lisbon60V [80]	- Widely used to characterize function of several ASFV genes [68], proteomic analysis [68,69], mechanisms of viral entry [72,73,74], transcription and replication [69,70] - Titration by plaque formation [65,66]	- Genomic mutation during adaptation → reduction of virulence and immunogenicity in pigs [61,67,79,80,81]
**COS**	African green monkey: *Chlorocebus sabaeus* [105]	From CV1 (see below) [105]	From CV1 (transformation with a mutant strain of Simian Virus 40 (SV40), which codes for the wild-type T-antigen) [105]	- E70 [82] - Malawi 82 [82] - Uganda [82] - Lisbon57 [82] - Lisbon60 [20,82] - Mozam68 [82] - CC83 [82]	- BA71V [20,30,82] - BA71ΔCD2 [81] - NH/P68 [82] - ΔEP153R [20,30,82]	- Used for production of large amount of virus [20], and studies on virus entry mechanisms [87,88] - Plaque titration [66,82] - Construction of deleted ASFV mutants [81,89] - BA71ΔCD2 –> stability and integrity in its genome [81] - BA71 → no changes in virulence and immunogenity [81]	- NH/P68 derived mutants → genomic mutations during passages relevant to protection [89]
**MS**	African green monkey [62,106]	Kidney [62,106]	Unknown process	- Low susceptibility to virulent to field isolate E70 [62]	- E70MS14, E70MS44, E70MS81 [62,63,90]		- Not plaque for titration [62] - Genomic mutation during adaptation → reduction of virulence and immunogenicity in pigs [62,90]
**CV1**	African green monkey: *Cercopithecus Aethiops* [107]	Fibroblast-like cells derived from kidney tissue [107]	Transformation with a mutant strain of Simian Virus 40 (SV40), which codes for the wild-type T-antigen [107]	- E75 [91] - Stavropol 01/80 [93]	- E75CV1 [91]	- Plaque titration - E75CV1 → 100% protection against challenge with homologous E75 in pigs [91] but not against heterologous BA71 [92].	- Stavropol 01/80 → Genomic mutation, during adaptation, with reduction of virulence and immunogenicity in pigs [93]
**Marc-145**	African green monkey Chlorocebus aethiops [94,108]	Fetal kidney epithelial cells, subpopulation of MA-104 [94,108]	MA-104 derived [94]	-D/ASF/POT/Vietnam/2019, D/ASF/POB/Vietnam/2019, although only three passages were monitored [95].			Unable to support the growth of ASFV Pol18/28298/Out111 [96] and ASFV-HLJ/18 [45].
**MA-104**	African green monkey: *Cercopithecus aethiops* [97,108]	Fetal kidney epithelial cells [97,108]	Spontaneously immortalized cell line [97]	- ASFV-G [97] - BA71 [97] - D/ASF/POT/Vietnam/2019, D/ASF/POB/Vietnam/2019 [95] - MW039157 [50]	- MW287337 [50]	- Suitable for ASFV isolation → able to detect ASFV with a TCID_50_ sensitivity comparable to that of primary swine macrophages [97] - Hemadsorption [97] - Genome stability during 15 passages of a genotype II ASFV isolates [98].	More studies required its suitability to grow ASFV strains for large-scale vaccine production.
**HEK293T**	Human [45]	Kidney epithelial [45]	Transformation with sheared Adenovirus 5 DNA [109]	- Low susceptibility to OURT88/3 [102] and ASFV-HLJ/18 [45]	- Adapted strain from ASFV-HLJ/18 (ASFV-P121) [45] - OURT 88/3-ΔTK-GFP [102]	- Efficiently and high replication of the attenuated virus [45] - Hemadsorption [45] - Clear cytopathic effect [45]	ASFV-HLJ/18 → Genomic mutation during adaptation (mainly at the MGF genes) [45] → potential reduction of virulence and immunogenicity in pigs [45]

Candidate ASFV LAVs passaged non porcine continuous cell lines and their impact in vivo are highlihted in red (promising results) or blue (unsucessful results). E70: España70; E75: España75; TCID_50_: 50% Tissue Culture Infectious Dose; and MGF: multiple multigene family.

## 6. Porcine Continuous Cell Lines

As previously stated, ASFV field isolates can be adapted to grow on established continuous cell lines through several culture passages; however, this adaptation is often accompanied by significant genetic modifications of the virus and the loss of immunogenicity [15,16,64]. The development of most vaccine candidates is indeed hindered by the lack of a continuous cell line that is adequately susceptible to ASFV while avoiding further genetic adaptations within the ASFV genome [15,16]; thus, several attempts have been made to establish a porcine cell line able to sustain ASFV growth.

Features, advantages and disadvantages of porcine continuous cell lines used for ASFV in vitro studies are summarized in Table 2.

### 6.1. PK Cell Lines

PK cells are porcine kidney cell lines, and different PK cell lines have been used to study ASFV: PK2a, PK9, PK0809, and PK15 [45,46,95,110,111]. Culture-adapted ASFV strains were able to induce cytopathic effects on PK cells, characterized by plaque formation (with plaque size heterogeneity) [110,112]. A plaque formation assay on this cell line showed a linear dose–response curve [112], and this method was sensitive and reproducible, although limited to culture-adapted ASFV strains. Nevertheless, COS-1 presently seems to be the most suitable cell line for plaque assay [30].

In the 1960s, several African, Portuguese, and Spanish ASFV isolates were passaged in PK2a. Some of these PK2a culture-adapted ASFV strains presented not only evident modifications in virulence when pigs were inoculated but also provided protection against challenges with a homologous virulent virus [110].

PK9 and PK0809 are other porcine kidney cells, and the former is characterized by the expression of porcine CD163 [113]. These cells were used by Lithgow et al. (2014) to investigate whether the expression of CD163 was correlated to the permissiveness to ASFV infection, as previously speculated by Sanchez-Torres et al. [35]. 

In the same study, researchers also investigated PK15 and created a cell line where exogenous porcine CD163 was stably expressed in PK15: PK15CD163. PK15, PK15CD163, PK9, and PK0809 were infected with several ASFV isolates: Benin 97/1, Virulent Uganda, BA71V, and Attenuated Uganda (using an MOI of 10), and researchers observed that the presence of CD163 did not influence the susceptibility of these cells to ASFV [46]. This indicated that CD163 alone is not sufficient for virus infection, in accordance with studies previously reported in this review (see Section 2.1).

In the same study, researchers observed that PK15, PK9, and PK0809 were susceptible to Attenuated Uganda and, to a lesser extent, to BA71V ASFV, whereas at 24 h pi with Benin 97/1 or Virulent Uganda few cells expressed early ASFV protein p30 [46]. We observed that at 24 h pi with either attenuated NH/P68 or virulent Sardinian 26544/OG10 (MOI = 1), almost undetectable levels of cells expressing late viral protein p72 were observed (Meloni and Franzoni, unpublished results).

PK15 cells were also used to screen some antibiotics in vitro, after adaptation of the ASFV Tengani strain by culture passage. That strain was adapted to culture by 45 passages on pig leucocyte cultures followed by 13 passages on PK15 and induced a clear cytopathic effect on these cells. Several rifamycin derivatives reduced the multiplication and the cytopathic effect of this ASFV strain on PK15 cells [111,114]. More recently, two studies suggested that PK15 cells are not suitable as a cell line to support ASFV replication, and better candidate cell lines were individuated in MA-104 [95] and HEK293T [45].

### 6.2. WSL Cells

WSL is a fetal wild boar lung cell line. These cells express high levels of SLA II and SW3 but almost undetectable levels of CD163 and CD169, suggesting that they represent immature precursors of macrophages [18,115]. Several ASFV strains, either field isolates or strains generated in the laboratory, successfully infected this cell line [20,30,87,116,117]. Subsequent studies have investigated the ability of WSL to efficiently support the growth of ASFV strains. 

In 2017, Sanchez and colleagues observed that this cell line sustained the growth of the attenuated NH/P68, whose production was higher in WSL cells compared to PAM. Nevertheless, WSL did not show efficient production of E70 or Armenia/07 [18,115]. More recently, two studies reported that WSL cells supported the growth of the virulent ASFV-Kenya1033-IX strain and of its derived mutant ASFV-KeΔA238L strain (generated by deletion of the gene A238L) [118,119]. ASFV-Kenya1033-IX reached levels in WSL similar to those obtained in PAMs when using an MOI of 1 [118]. 

At the genomic level, Hemmil and colleagues observed only a few single nucleotide differences between ASFV-Kenya1033-IX grown in macrophage or passaged in WSL [118], and Abkallo and colleagues confirmed the stability of the virus in this cell line; no SNPs, deletions, or rearrangement were observed in ASFV-Kenya1033-IX and its derived mutant ASFV-KeΔA238L replicating in WSL [119]. In addition, both NH/P68 and ASFV-Kenya1033-IX passaged several times in WSL were still able to infect and replicate in both primary macrophages and pigs; all of these studies are paving the way for the development of an ASFV-LAV in the future [18,118].

Quantitative mass spectrometry was employed to investigate gene expression of ASFV in WSL and other cell lines. Analyses showed that the expression profiles of OUTR88/3-infected WSL differed remarkably form those of OUTR88/3-infected Vero and HEK293T, although some similarities were observed between WSL and Vero [102]. In a more recent study, quantitative mass spectrometry was employed to compare the proteome of the porcine moMΦ and WSL after infection with the ASFV isolate Kenya1033. 

The expression profile of viral proteins was very similar in both cell lines, although cell-specific expression of some individual viral proteins did occur [120]. The authors concluded that both infection models were suitable for investigating ASFV infection biology. Nevertheless, a larger number of genes were downregulated in moMΦ compared to WSL cells, suggesting that ASFV induces a host shut-off in moMΦ but no or only a weaker shut-off in WSL cells. This aspect should be taken into account in host interaction studies involving WSL [120].

Overall, these studies suggested that WSL cells are suitable for the production of attenuated and highly virulent ASFV virus strains [18,118] and as an alternative method to study ASFV infection biology; nevertheless, they cannot be used for titration because the cytopathic effects are slight or unnoticeable [20,30]. This cell line can be a cell model for the development of antiviral protection using the CRSPR-Cas9 method [121].

### 6.3. Immortalized Porcine Alveolar Macrophages

Immortalized porcine alveolar macrophages (IPAM) is a continuous cell line derived from porcine alveolar macrophages. The most popular IPAM cell line is 3D4, generated by the transfection of primary PAMs with plasmid pSV3neo, carrying genes for SV40 large T antigen. Several clones were obtained following PAM transfection, which were subsequently grown in a positive selection pressure medium for 14 weeks. Among these clones, 3D4 was selected. Single-cell cloning of the 3D4 parent led to establishing diverse cell lines, including 3D4/2, 3D4/21, and 3D4/31 [122]. These cells present several differences from PAMs: they express low percentages of CD14 and SW3 (5.5 and 28%, respectively) and almost undetectable levels of CD163 and SLA II; thus, they do not present a mature macrophage phenotype [18,30].

The ability of 3D4/2, 3D4/21, and 3D4/31 to sustain the growth of the cell-culture-adapted ASFV-Lisbon 61 and the field isolate Lillie SI/85 was monitored. All the tested cell lines supported the replication of the adapted ASFV-Lisbon 61 and the isolate Lillie SI/85 but to different degrees. Researchers observed that ASFV-Lisbon 61 grew more efficiently in 3D4/2, 3D4/21, and 3D4/31, compared to the isolate Lillie SI/85. Interestingly, the ability of this cell line to support infection was linked to the formulation of the culture medium: the addition of DMSO in the 3D4/21 medium improved its ability to support ASFV/Lillie replication [122]. 

More studies are needed to determine the factors underlying the DMSO-mediated increased susceptibility to infection of 3D4/21. It was later described that IPAM was sensitive to two ASFV isolates (Hinde att and Uganda att), whereas infection with other ASFV strains resulted in weak PCR results or infective progeny production (BA71V, NH/P68, Malawi82, and Lisbon57) [30].

In 2017, Sanchez and collaborators determined that IPAM was not susceptible to infection with either virulent Armenia/07 or attenuated NH/P68 (almost undetectable levels of late viral protein p72 were detected by flow cytometry at 18, 40, and 72 h pi) [18,115]. In addition, it was more recently determined that 3D4/21 was unable to maintain replication of the ASFV-HLJ/18 strain (no ASFV genome copy numbers were detected at passage 5) [45]. 

### 6.4. Immortalized Porcine Kidney Macrophages

Immortalized porcine kidney macrophages (IPKM) is an established cell line isolated from primary porcine kidney-derived macrophages (PKMs). The primary PKM cell line was immortalized by transfecting recombinant lentivirus vectors carrying the gene for SV40 large T antigen (SV40LT) in combination with the gene for porcine telomerase reverse transcriptase (pTERT) [123]. 

The derived continuous cell lines presented a morphology similar to that of primary macrophages, expressed specific macrophage surface markers (Iba1, KT022, and CD172a), and were negative for epithelial (CK18 and CK19) and mesenchymal (SMA) cell markers [123]. The levels of phagocytosis of IPKM and PKM cells were similar; in addition, IPKM released pro-inflammatory cytokines (TNFα and IL-1β) in response to lipopolysaccharide stimulation to the same extent as porcine PKM [123].

The susceptibility of this continuous cell line to ASFV was subsequently investigated. This cell line has shown high susceptibility to different virulent field ASFV isolates: Armenia07 (genotype II), Kenya05/Tk-1 (genotype X), and E75 (genotype I) and also to the VERO cell-adapted isolate Lisbon60V (genotype I) [23]. Infection of IPKM with Armenia07, Kenya05/Tk-1, and E75 resulted in a clear cytopathic effect; rosette formation was also observed with a HAD assay. A plaque assay revealed that ASFV infection determined the formation of clear plaques, useful for a rapid isolation and purification of different strains. In addition, viral titers produced by all tested ASFV strains in IPKM were very similar to those of PAM cells. 

Most importantly, next generation sequencing revealed a good genomic stability of Armenia07 after 5, 10, and 15 passages on IPKM, with only one non-synonymous nucleotide replacement detected in the CP530R region observed at passages 10 and 15 [23]. Overall, these results suggested that the IPKM line could be used for isolation, propagation, and manipulation of ASFV; however, further studies are required.

### 6.5. Plum Island Porcine Epithelial Cells

Plum Island porcine epithelial cells (PIPEC) are a stable cell line derived after 60 passages from the LFPKaVb6 (a porcine fetal kidney cell line engineered to express a bovine integrin [124]). PIPEC was investigated as a putative cell line for the large-scale production of ASFV-G-ΔI177L, a candidate ASFV vaccine created by deleting the I177L gene from the highly virulent ASFV-G [125]. ASFV-G-ΔI177L was a promising candidate LAV owing to its safety and ability to protect against challenge with parental ASFV-G, although it was able to replicate only in primary macrophages, which cannot be used for large-scale vaccine production, as previously mentioned in this review. 

ASFV-G-ΔI177L was passaged in PIPEC, and this adaptation resulted in the generation of a new strain (ASFV-G-ΔI177LΔLVR) characterized by a deletion of 10,842 in the left variable region (LVR). ASFV-G-ΔI177LΔLVR efficiently replicated in PIPEC, and even after 30 passages, no additional genomic changes were observed. ASFV-G-ΔI177LΔLVR presented a phenotype and safety profile similar to the parental ASFV-G-ΔI177L and replicated efficiently in swine primary macrophages. Most interestingly, ASFV-G-ΔI177LΔLVR protected against challenge with the highly virulent ASFV-G. Overall, the data suggested that PIPEC is a suitable cell line for the large-scale production of a safe and commercial vaccine [125].

### 6.6. Zuckerman Macrophage-4

The Zuckerman macrophage-4 (ZMAC-4) is a pig macrophage cell line derived from lung macrophages of a porcine fetus [126]. These cells are maintained in culture by adding 20 ng/mL of M-CSF to culture media, and they express several surface markers characteristic of PAMs, such as CD14, CD163, CD172, and E26-family transcription factor PU.1. Nevertheless, ZMAC-4 cells did not express CD203a, suggesting that they are less mature than primary cells. In addition, ZMAC-4 cells can proliferate; after 34 days, a >100-fold expansion from the initial cell number was achieved. The ZMAC-4 cells form colonies suspended in the medium [126]. Not only do ZMAC-4 cells have many characteristics similar to PAMs, they can also support the growth of the monocytropic PRRSV [127].

Recently, it was observed that ZMAC-4 cells were also highly susceptible to ASFV infection. These cells were susceptible to infection with either virulent (Benin 1997/1, Georgia 2007/1, Malawi LIL20/1, Tengani, MOZ 94/1, ZOM 2/84, Dominican Republic) or attenuated (OUTR88/3, NH/P68) ASFV strains, to levels similar to those of bone-marrow-derived macrophages. In addition, ZMAC-4 cells efficiently supported the growth of Georgia 2007/1 to levels similar to bone-marrow-derived macrophages. Finally, the authors observed that 12 passages of the attenuated OURT88/3 ASFV in ZMAC-4 cells did not modify its safety and did not reduce its ability to induce a 100% protective response in pigs against challenge with virulent ASFV, demonstrating the efficacy of vaccine strains produced in this cell line [126]. 

All these results confirmed that ZMAC-4 cells provide a realistic alternative to primary macrophages, indicating it to be a suitable cell line for research on ASFV.

### 6.7. A_4_C_2_ and A_4_C_2_/9k Cells

A_4_C_2_ and A_4_C_2_/9k are hybrid cell lines of SPEV TK with swine lymphocytes and were used to adapt the Russian ASFV strain Stavropol 01/08. Both epitheliocytes and lymphocyte-like cells were present in A_4_C_2_, whereas A_4_C_2_/9k presented an increased content of lymphocyte-like cells [93]. Adaptation was performed with several passage methods. Stavropol 01/08 was adapted first to A_4_C_2_; an increase in the ASFV virus titer was observed at passage 7, and the strain was fully adapted to A_4_C_2_ at passage 11 when the titer remained stable.

The adapted strain also grew efficiently in A_4_C_2_/9k cell culture. Other isolates that multiplied in this cell line were Sveromorsk 2010, Volgograd/Kalach 2012, Tver/Zavidovo 2012, etc. Nevertheless, the virus titer determined in the A_4_C_2_/9k cell culture was 0.7–1.25 log lower than that in the peripheral blood monocyte cell culture [93]. Interestingly, in both cell lines, hemadsorption was visible after adding 0.5% suspension of pig erythrocytes [93].

Culture variants of strain Stavropol at passage 14 in the A_4_C_2_/9k maintained the proper virulent titers, whereas at passages 24 and 33, the strain lost its pathogenicity for pigs. Nevertheless, both passages 24 and 33 did not confer protection against challenge with parental virulent strain [93].

### 6.8. PSGK-60 and PPK-66b

PSGK-60 and PPK-66b are continuous pig kidney cell lines, where several virulent Russian isolated ASFV strains were adapted [93]. In these cells, ASFV Stavropol 01/08 was passaged, and around passage 14, the strain was adapted to both cell lines. In addition, researchers observed that Stavropol 01/08 culture variants at passage 20 in the PSGK-60 line maintained virulent properties; infected pigs died with an acute form of the ASFV disease [93]. No hemadsorption was observed in either cell line [93].

The PPK-66b cell line was also used to generate attenuated ASFV strains from both TSP-080 and TS-7 (two ASFV strains derived from ASFV Kiravira-67). TSP-080/300 (derived from virulent TSP-080) and TS-7/230 (derived from TS-7) were both low-reactogenic and able to protect pigs against challenge with the corresponding parental strain. In addition, the Virulent Uganda ASFV was passaged in PPK-66b, and the derived strain (UK-50) was characterized by “loose” hemadsorption [128].

Candidate ASFV LAVs passaged porcine continuous cell lines and their impact in vivo are highlighted in red (promising results) or blue (unsuccessful results). E70: España70; IPAM: immortalized alveolar macrophages; SV40: Simian Virus 40; IPKM: immortalized porcine kidney macrophages; pTERT: porcine telomerase reverse transcriptase; E75: España75; PIPEC: Plum Island porcine epithelial cells; and ZMAC-4: Zuckerman macrophage-4.

**Table 2:** Characteristics of porcine-derived continuous cell lines used for ASFV in vitro studies and the ability to support the growth of ASFV candidate vaccines.

Cell Line	Species of Cell Origin	Tissue of Cell Origin	Mechanism of Immortalization	Susceptibility to Field Isolates	Susceptibility to Adapted Isolates	Advantages	Disadvantages
**PK**	Pig [110,112,129]	Kidney [110,112,129]	Spontaneously immortalized cell line [129]	- PK2a: extremely low susceptibility to several ASFV strains: Spencer, Portuguese, Gasson, Madrid n. 1, Madrid n. 2 [110] - PK/A/C/13: extremely low susceptibility to Uganda and Hinde: [112] - PK15: extremely low susceptibility to Tengani [111,114]	-PK2a: susceptibility to ASFV strains after several passages on PK2a (Spencer, Portuguese, Gasson, Madrid n. 1, Madrid n. 2) [110] - PK/A/C/13: susceptibility to Uganda and Hinde after several passages on PK cells [112] - PK15, PK9, and PK0809: Attenuated Uganda, BA71V [46]. - PK15: strain derived from Tengani through passages on PK15 [111,114]	- Plaque titration (with plaque size heterogeneity) [110,112]. - Clear cytopathic effects [110] - Hemadsorption [110]	- Plaque formation limited to culture-adapted ASFV strains [30,110]. - PK15 no able to support the growth of ASFV-HLJ/18 strain [45], Benin 97/1, Virulent Uganda [46]. - PK9, and PK0809 no susceptible to Benin 97/1, Virulent Uganda [46]. - PK15 no able to support the growth of D/ASF/POT/Vietnam/2019, D/ASF/POB/Vietnam/2019 [95] - Spencer, Portuguese, Gasson adapted to PK cells → genomic mutation during adaptation with reduction of virulence in pigs but only partial protection against challenge with homologous strain [110] - Hinde isolates passaged 75 times in PK2a → reduction of virulence in pigs but only partial protection to challenge to parental strain; survived pigs only partial protection to other ASFV isolates [65]
**WSL**	Wild boar (fetus) [20,30,116]	Lung [20,30,116]	Spontaneously immortalized cell line [130]	- CC83, Malawi 82, Uganda, Lisbon 57, E70 [30] -ASFV-Kenya1033-IXL [118]	- NH/P68 [18,115] - ΔEP153R, Hinde attenuated, Uganda attenuated NH/P68, BA71V [30] - NHV-dTK-EGFP [116] - ASFV-KeΔA238L strain (generated by deletion of the gene A238 by ASFV-Kenya1033- IXL) [119] - OUTR88/3 [102]	- ASFV-Kenya1033-IXL and ASFV-KeΔA238L → genomic stability during passages in WSL [118,119] - NH/P68 ASFV strain produced in WSL cells (10 passages) → in vivo in pigs induced a similar infection pattern to that of NH/P68 passaged in PAM [18,115] - ASFV-Kenya-1033-IX produced in WSL cells (more than 20 passages) → retained the virulence in vivo [118]	- Slight or unnoticeable cytopathic effects [20,30] - No ability to efficiently support the growth of E70 or Armenia/07 [18,115]. - Promising results, but in vivo studies are required to evaluate its suitability to grow ASFV strains for large-scale vaccine production (protection to challenge).
**IPAM**	Sus scrofa [30]	Porcine alveolar macrophages [30,122]	Porcine myeloid cell lines established by transfecting primary porcine alveolar macrophage cultures with plasmid pSV3neo, carrying genes for SV40 large T antigen [30,122]	- Low susceptibility to few virulent isolates, including E70, CC83 [30] - Low susceptibility to field isolateLillie SI/85 [122]	- Uganda attenuated, Hinde attenuated [30] - 3D4/2, 3D4/21, and 3D4/31: growth of the attenuated ASFV-Lisbon 61 supported in different degrees. [122]		- Unable to maintain replication of the ASFV-HLJ/18 strain [45] - Weak susceptibility to BA71V, NH/P68, Malawi82, Lisbon57 [30]. - No susceptible to infection with Armenia/07 or NH/P68 [18,115].
**IPKM**	Pig [123]	Primary porcine kidney-derived macrophages [123]	Immortalization by transfection with recombinant lentivirus vectors carrying the gene for SV40 large T antigen (SV40LT) in combination with the gene for porcine telomerase reverse transcriptase (pTERT) [123]	- Armenia07 [23] - Kenya05/Tk-1 [23] - E75 [23]	- Lisbon60V (VERO cell-adapted isolate) [23]	- Clear cytopathic effect [23] - Hemadsorption [23] - Plaques formation → rapid isolation and purification of different strains [23] -Armenia07 → good genomic stability with only one non-synonymous nucleotide replacement detected in the CP530R region at passages 10 and 15 [23]	Promising results, but in vivo studies are required to evaluate its suitability to grow ASFV strains for large-scale vaccine production.
**PIPEC**	Pig (fetus) [124]	Kidney [124]	60 passages from the LFPKaVb6 (a porcine fetal kidney cell line [124]).		- ASFV-G-ΔI177L → generation of ASFV-G- ΔI177LΔLVR after several passages [125]	- ASFV-G- ΔI177LΔLVR efficiently replicated in PIPEC and after 30 passages no additional genomic mutation [125]. - ASFV-G-ΔI177LΔLVR → safe and protective (100% protection against challenge with parental virulent ASFV-G) [125].	Promising results, but more studies are required to evaluate its suitability to grow ASFV strains for large-scale vaccine production.
**ZMAC-4**	Pig (fetus) [126]	Lung macrophages [126].	Spontaneously immortalized cell line [126].	- Benin 1997/1, - Georgia 2007/1, - Malawi LIL20/1, - Tengani, - MOZ 94/1, - ZOM 2/84, - Dominican Republic [126]	- OUTR88/3 - NH/P68 [126]	- Efficiently supported the growth of Georgia 2007/1 to levels similar to bone-marrow-derived macrophages [126]. - 12 passages of the attenuated OURT88/3 ASFV in ZMAC-4 cells → safe and protective (able to induce a 100% protective response in pigs against challenge with virulent ASFV) [126]	Promising results, but more studies are required to evaluate its suitability to grow ASFV strains for large-scale vaccine production.
**A_4_C_2_ and A_4_C_2_/9k**	Pig [93]	Hybrid cell lines of SPEV TK with swine lymphocytes [93]	Not described [93]	-Stavropol 01/08 [93] - Sveromorsk 2010, Volgograd/Kalach 2012, Tver/Zavidovo [93]		- Hemadsorption was visible after adding 0.5% suspension of pig erythrocytes [93].	- Stavropol at passage 14 in the A4C2/9k maintained the proper virulent titers strain [93] - Stavropol at passages 24 and 33 → loss of pathogenicity in pigs but no protection to challenge with parental virulent strain [93].
**PSGK-60/PPK-66b**	Pig [93]	Kidney [93]	Not described [93]	- Stavropol 01/08 in PSGK-60 and PPK-66b [93] - TSP-080 and TS-7 (two ASFV strains derived from ASFV Kiravira-67) in PPK-66b [128] - Uganda in PPK-66b [128]	- TSP-080/300 (derived from TSP-080) in PPK-66b [128] - TS-7/230 (derived from TS-7) in PPK-66b [128] - Uk-50 (derived from Uganda): PPK-66b [128]	- Stavropol 01/08: maintained virulent properties → infected pigs died [93] - TSP-080/300 and TS-7/230 →Low-reactogenic and able to protect pigs against challenge with the corresponding parental strain [128].	- No hemadsorption [93] -Promising results, but more studies are required to evaluate its suitability to grow ASFV strains for large-scale vaccine production.

## 7. Conclusions

**Figure 3:**
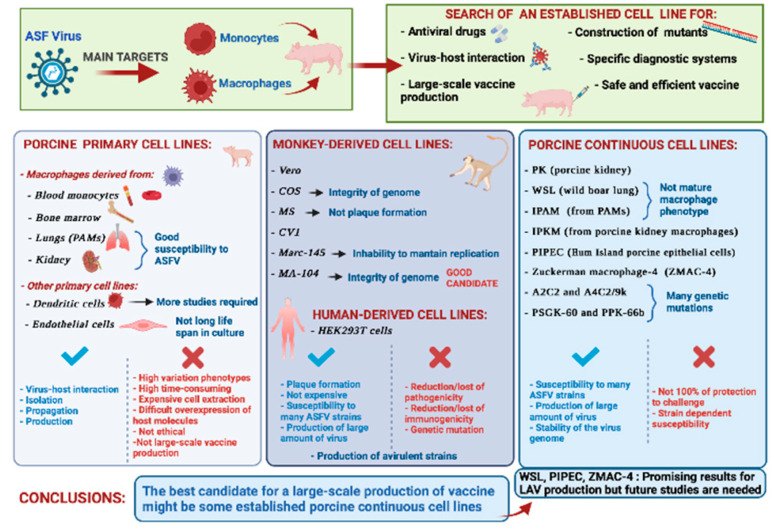
Primary and continuous cell lines tested for ASFV susceptibility. The main targets of ASFV are porcine monocytes and macrophages. Established cell lines were tested to support virus growth for the production of antiviral drugs, virus–host interaction studies, the construction of mutants, specific diagnostic systems, and the large-scale production of a safe and efficient vaccine. The established cell line has to have susceptibility to several ASFV strains, be not expensive to produce or time-consuming, be ethical, and able to maintain the genome stability of the virus. Considering all the cells tested thus far, the most promising continuous cell lines for LAV production are WSL, PIPEC, and ZMAC-4; however, future studies are still needed. Created with BioRender.com (accessed on 7 March 2022). PAMs: porcine alveolar macrophages; IPAM: immortalized porcine alveolar macrophages; IPKM: immortalized porcine kidney macrophages; PIPEC: Plum Island porcine epithelial cells; ZMAC-4: Zuckerman macrophage-4; and LAV: live attenuated vaccine.

In this review, we summarized the research on the most suitable cell lines for ASFV isolation and propagation to facilitate not only in vitro studies but also the development and large-scale production of a vaccine against ASFV (Figure 3).

For many years, macrophages were used to isolate ASFV from field outbreaks and to investigate the infection biology of this virus; nevertheless, obtaining primary macrophages is time-consuming and expensive, and large-scale vaccine production using these primary cells is not feasible and is ethically fraught. These obstacles have been partially overcome by the adaptation of virulent field isolates on monkey or human continuous cell lines; however, several culture passages often resulted in significant genetic modifications of the passaged virus and the loss of immunogenicity. 

Future studies are needed to better understand genome mutations due to adaptation that leads to the loss of immunogenicity. Only two monkey-derived cell lines (COS and CV1) were able to support the growth of safe and efficient LAVs; nevertheless, not all the attenuated strains tested were able to confer protection to challenges with virulent isolates (Table 1). Thus, several attempts have been made to establish a porcine cell line that is able to sustain ASFV growth. 

Preliminary data indicate that some porcine continuous cell lines might be realistic alternatives to primary macrophages for research on ASFV and for the large-scale production of a vaccine against this virus. Thus far, promising candidate cell lines for ASFV LAVs were individuated in WSL, PIPEC, ZMAC-4, PPK-66b, which supported the replication of NH/P68, ASFV-G-ΔI177L, OURT88/3, TSP-080/300, and TS-7/230, respectively (Table 2). Although the preliminary results on some porcine cell lines are promising, further studies are required for the use of these cell lines for the safe large-scale production of ASFV vaccines. 

## Data Availability

Not applicable.
